# Natural Killer Cell Recognition of Melanoma: New Clues for a More Effective Immunotherapy

**DOI:** 10.3389/fimmu.2015.00649

**Published:** 2016-01-07

**Authors:** Raquel Tarazona, Esther Duran, Rafael Solana

**Affiliations:** ^1^Immunology Unit, University of Extremadura, Caceres, Spain; ^2^Histology and Pathology Unit, Faculty of Veterinary Medicine, University of Extremadura, Caceres, Spain; ^3^Immunology Unit, Instituto Maimónides de Investigación Biomédica de Córdoba (IMIBIC), Reina Sofia University Hospital, University of Cordoba, Cordoba, Spain

**Keywords:** melanoma, immunotherapy, natural killer cells, adoptive transfer, checkpoint blockade

## Abstract

Natural killer (NK) cells participate in the early immune response against melanoma and also contribute to the development of an adequate adaptive immune response by their crosstalk with dendritic cells and cytokine secretion. Melanoma resistance to conventional therapies together with its high immunogenicity justifies the development of novel therapies aimed to stimulate effective immune responses against melanoma. However, melanoma cells frequently escape to CD8 T cell recognition by the down-regulation of major histocompatibility complex (MHC) class I molecules. In this scenario, NK cells emerge as potential candidates for melanoma immunotherapy due to their capacity to recognize and destroy melanoma cells expressing low levels of MHC class I molecules. In addition, the possibility to combine immune checkpoint blockade with other NK cell potentiating strategies (e.g., cytokine induction of activating receptors) has opened new perspectives in the potential use of adoptive NK cell-based immunotherapy in melanoma.

## Introduction

Melanoma is largely resistant to current therapies as chemotherapy and radiotherapy (1) and consequently remains as an important cause of mortality mainly in Caucasians. Metastatic melanoma is highly aggressive constituting the most lethal skin cancer (2). Despite the different approaches developed for primary prevention of melanoma, its incidence rate continues increasing in many countries (3).

It has been postulated that melanoma ability of inducing an immune response contributes to patient survival. Thus, melanoma is usually highly immunogenic and induces cytotoxic T cell (CTL)-mediated immune responses. Tumor infiltrating lymphocytes (TILs) have been identified in melanoma lesions usually associated with spontaneous tumor regression and favorable prognostic in primary melanoma (4).

Innate immune responses against melanoma have also been described. Natural killer (NK) cells constitute the first line of defense against transformed cells as tumors or virus-infected cells. *In vitro* experiments have established that NK cells can recognize and destroy melanoma cell lines (5–7). The role of NK cells against melanoma *in vivo* has been demonstrated in murine models (8), and it is also supported by the observation of NK cell alterations (e.g., down-regulation of activating receptors or NK cell exhaustion) in melanoma patients (9, 10) suggesting the development of escape mechanisms to evade NK cell-mediated destruction of melanoma cells.

It is well known that age affects both adaptive and innate immune responses against tumors (11–14). The hypothesis of immunosurveillance against melanoma is further sustained by the recent finding that elderly melanoma patients had a higher incidence of melanoma-related mortality than younger patients in spite of the lower incidence of sentinel lymph node metastasis (15).

Altogether, these characteristics of melanoma reinforce the previous consideration of melanoma as a suitable model for studying tumor immunity. Here, we review the current state of knowledge on NK cell-mediated recognition and lysis of melanoma cells and the up to date immunotherapeutic strategies against melanoma based on NK cells.

### NK Cell-Mediated Anti-Melanoma Responses

The key role played by NK cells as a first line of defense against tumors has been established in hematological malignancies based on the graft-versus-leukemia effect (16–18). However, their role against solid tumors such as melanoma is less recognized. It has been reported that NK cells contribute to melanoma surveillance *in vivo* (19–21). NK cells can actively participate in the initial phase of tumor development and may control metastasis, but the direct action of NK cells against tumor tissue is not well known. NK cells may contribute to cancer elimination not only by the lysis of tumor cells but also by the secretion of cytokines and the promotion of antigen-presenting cell maturation contributing to the adaptive immune response (22–24).

**Figure 1:**
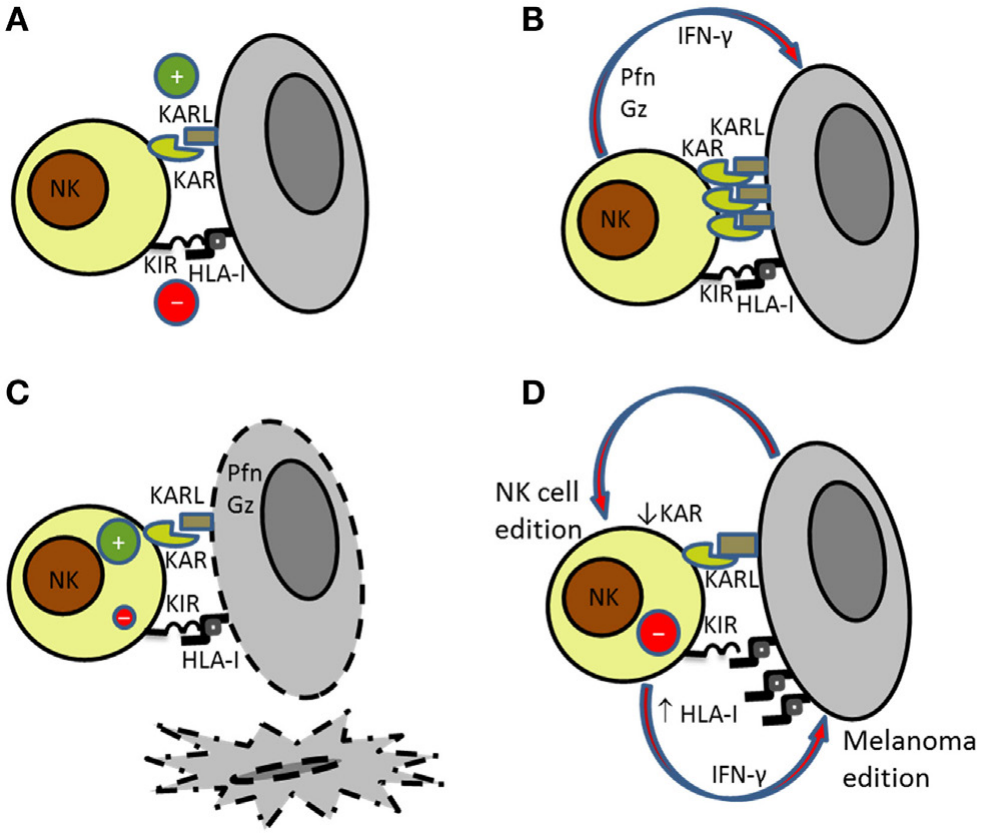
**Bidirectional interaction of NK cells with melanoma cells**. **(A)** NK cell recognition of targets depends on the balance between activating (KAR) and inhibitory signals (KIR). **(B)** Activated NK cells secrete perforin (Pfn) and granzymes (Gz) that are involved in **(C)** NK cell-mediated killing of susceptible targets. **(D)** Melanoma cells became resistant to NK cell-mediated killing by increasing the expression of HLA class I molecules. At the same time, NK cells reduce the expression of activating receptors further contributing to melanoma escape.

Natural killer cells express several activating receptors that after cross-linking with their respective ligands trigger NK cell degranulation releasing their cytotoxic granule content leading to target cell apoptosis (Figure 1A). Research during the last decade has highlighted that several activating receptors are involved in NK cell recognition of tumor cells (6, 25). The existence of diverse ligand–receptor interactions is relevant in melanoma recognition since it has been demonstrated that melanoma cells express a variety of ligands for different NK cell-activating receptors (7). It has been postulated that the integration of multiple activating signals may overcome the inhibitory signals mediated by major histocompatibility complex (MHC) class I-specific inhibitory receptors (25, 26). In addition, different ligands may interact with the same activating receptor as occur for NKG2D ligands (MICA/B and ULBPs) (27) and DNAM-1 ligands [CD112, also named Nectin-2, and CD155 that is considered the poliovirus receptor (PVR)] contributing together to NK cell activation (28). Recently, the family of receptors that bind nectin and nectin-like proteins has expanded. It has been described that some of these activating receptors have an inhibitory counterpart that compete for the same ligands. For instance, the activating DNAM-1 and the inhibitory T cell immunoreceptor with immunoglobulin and ITIM domains (TIGIT) compete for the same ligand (CD155) on the target cells, regulating NK cell activation (29). The receptor TACTILE (CD96) also binds CD155 and may inhibit cytokine secretion in mice (30, 31), although its role in human NK cell function remains unclear. Other receptor for nectin-like proteins is CRTAM that is expressed on NK cells and CD8 T cells upon activation and binds nectin-like 2 promoting adhesion to target cells (32).

A characteristic that makes melanoma a prototype for the study of NK cell-mediated tumor destruction is the fact that melanoma cells frequently show altered expression of MHC class I molecules (33). Diminished expression of MHC class I molecules makes melanoma cells unaffected by CTLs but facilitate NK cell killing (34). The altered MHC class I phenotypes on tumor cells can be classified as reversible (“soft lesions”) when the MHC class I expression can be recovered or upregulated after cytokine treatment or irreversible (“hard lesions”) when the molecular defect is structural and cannot be recovered such as loss of heterozygosity due to mutations on β2 microglobulin (34). Thus, the molecular mechanisms involved in the down-regulation or loss of MHC class I molecules in tumor cells have an impact on tumor development and in CTL-based immunotherapy efficacy. In experimental and clinical models, tumor regression has been associated with reversible MHC class I alterations whereas irreversible alterations were linked with tumor progression (34–36).

Mature NK cells express CD16 (FcγR-III) that mediates antibody-dependent cell cytotoxicity (ADCC) representing an effective mechanism of lysis of antibody-coated target cells. However, it has been described that NK cell activation is associated with metalloproteinase-mediated cleavage of CD16 molecules. The treatment with metalloproteinase inhibitors prevented CD16 down-regulation and increased NK cell polyfunctionality (cytokine production and degranulation). The use of metalloproteinase inhibitors in monoclonal antibody (mAb)-based immunotherapy is proposed to benefit cancer patients (37).

### Melanoma Cells Express Ligands for NK Cell-Activating Receptors

We have previously analyzed a large panel of melanoma cell lines from the “European Searchable Tumor Cell Line and Data Bank” (ESTDAB, http://www.ebi.ac.uk/ipd/estdab/) and “Outcome and impact of specific treatment in European research on melanoma” (OISTER, QLG1-CT-2002-00668) projects demonstrating a high expression of ligands for NK-cell activating receptors on these cell lines. A high percentage of melanoma cell lines expressed ligands for NKG2D (85%) and DNAM-1 (95%)-activating receptors. The expression of MICA/B on melanoma cell lines prevailed over ULBP expression (7). Several studies have analyzed the expression of NKG2D ligands on melanoma specimens by immunohistochemistry showing a high heterogeneity. MICA/B expression was observed at a higher frequency than ULBP2 on melanoma metastasis (38). The analysis of MICA expression on melanoma lesions revealed a higher expression in primary melanoma than in metastatic melanoma (39, 40). The pattern of expression was not homogeneous, and interestingly, in some patients, a preferential staining was observed at the invasive front (38). Regarding DNAM-1 ligands, CD155 was found to be expressed in the majority of melanoma cell lines analyzed in contrast with the 26% of melanoma cell lines expressing CD112 (7). The expression of CD155 on melanoma specimens and melanoma cell lines also showed a stronger expression on metastatic melanoma compared to primary melanoma (41).

The identification of cellular ligands for the natural cytotoxicity receptors (NCRs) NKp30, NKp44, and NKp46 has remained elusive until recently. The use of chimera proteins constructed using the extracellular domain of NKp30, NKp44, or NKp46 fused to the Fc immunoglobulin domain (NCR-Fc) or to an amino-terminal isoleucine zipper (NCR-ILZ) allowed to analyze the expression of NCR ligands on tumor cells. A high variability in the binding of NCR chimeras to melanoma cells was observed with melanoma cell lines expressing ligands for NKp30 and NKp44 but not for NKp46 (6, 42) and other cell lines expressing ligands for NKp46 (43). The study of melanoma lesions in patients with metastatic tumors identified NKp44 ligands in all melanoma samples analyzed and NKp30 ligands in the majority of samples, whereas the expression of NKp46 ligands was null (44). The expression of NCR ligands was also analyzed on melanoma cells from lymph nodes and paired samples obtained from skin metastasis. Melanoma cells from lymph nodes showed staining with NKp44-Fc and NKp46-Fc chimeras and were more susceptible to NK cell-mediated lysis than melanoma cells from skin metastasis that had low or negative staining with NCR-Fc (6). These differences probably represent different stages of the disease. Thus, it has been proposed that in early stages, melanoma cells overexpress NCR ligands and during melanoma progression NCR ligand expression is down-regulated (6, 43) representing an immunoescape mechanism used by melanoma.

Recently, several cellular ligands for NCRs have been identified. NKp30 recognizes B7-H6 that has been found expressed on melanoma cell lines (45), human leukocyte antigen (HLA)-B-associated transcript 3 (BAT3) (46), and CMV pp65 tegument protein (47). The proliferating cell nuclear antigen (PCNA) has been recognized as a NKp44 ligand (48, 49). In contrast, cellular ligands for NKp46 remain elusive. The characteristics of NCR ligands identified so far suggest that these receptors may recognize damage-associated molecular patterns related to cellular stress (e.g., tumor transformation or infection) (50). *In vitro* receptor blocking experiments showing NCR-mediated lysis of melanoma cell lines further support the role of this receptor family in the control of melanoma (5, 6, 51).

### NK Cell–Melanoma Interaction

Natural killer cell recognition and lysis of melanoma cells involve different receptor–ligand interactions including NKG2D-, DNAM-1-, and NCRs-activating receptors. The expression pattern of ligands for activating receptors on melanoma and the expression of MHC class I molecules recognized by inhibitory receptors will determine the activation of NK cells (Figures 1A–C). As indicated before, NK cell lysis of melanoma cells may depend on the disease stage and the anatomical location due to the differential expression of ligands (6, 52). Antibody blocking experiments have demonstrated that usually melanoma cell lysis requires signaling through several activating receptors (25, 52).

The role of NKG2D in NK cell recognition and lysis of melanoma cells has been extensively discussed. Whereas, NKG2D is clearly involved in the lysis of melanoma cells expressing high levels of NKG2D ligands, and NCRs and DNAM-1 are the receptors involved in the elimination of melanoma cells with low expression of ligands for NKG2D. Thus, it has been described that NCRs and DNAM-1 cooperation is frequently involved in the lysis of melanoma cells both in humans and in mice (6, 53). The participation of several activating receptors in the activation of NK cells against melanoma contributes to the effective NK cell-mediated lysis of these cells (Figure 1).

The majority of studies analyzing effector–target interactions in melanoma are performed using cell lines cultured as monolayer or in suspension testing ligand expression correlation with CTL- or NK cell-susceptibility to lysis. Recently, the use of three-dimensional (3D) cell culture systems has been proposed for the analysis of melanoma interaction with lymphocytes. Thus, melanoma cells grown in 3D architecture showed lower recognition by melanoma-specific CTLs compared to those melanoma cells growing in 2D monolayers. It has been proposed that culture in 3D affects the expression of molecules involved in melanoma recognition by CTLs (54, 55). We can speculate that 3D culture also alter the expression of ligands for NK-cell activating receptors increasing melanoma resistance to NK cell lysis in a similar way as occurs in melanoma tissue.

An expansion of highly cytotoxic CD57^+^ NK cells has been found in tumor-infiltrating lymph nodes in melanoma patients. Their potential use as a source of cytotoxic NK cells for adoptive immunotherapy is discussed (56). The expansion of highly mature CD57^+^ NK cells has been observed in CMV-seropositive individuals, and it is further increased by age (11, 12). These cells represent highly differentiated NK cells with low proliferative capacity and high cytotoxicity. Although these cells have a lower expression of NKp30 and NKp46 (57–59), the expression of the activating receptors DNAM-1 and NKG2C is increased on the CD57^+^ subpopulation of CD56^dim^CD16^+^ NK cells in CMV-seropositive young donors, but it is reduced in the old individuals (59). These changes in the expression of cytotoxicity activating receptors may have functional relevance not only against CMV infection but also against other age-associated diseases as cancer. Thus, the potential use of CD57^+^ NK cells in melanoma immunotherapy requires a detailed analysis of their cytotoxic capacity and the expression of activating receptors since it depends on other factors as CMV latent infection and age (11, 12, 59).

### Checkpoints in NK Cell Activation

Natural killer cell activation depends on a tune balance mediated by inhibitory and activating signals transmitted through surface receptors upon contact with their respective ligands. In this process, the interaction between MHC class I molecules on target cells and MHC class I-specific inhibitory receptors on NK cells represents a major checkpoint regulating NK cell functions (60). Killer cell immunoglobulin-like receptors (KIR) are a family of highly polymorphic receptors that recognize MHC class I molecules. Inhibitory and activating KIRs have been described. KIRs govern NK cell education and function and inhibitory KIR–HLA interactions may be associated with failed tumor immunosurveillance mediated by NK cells (61). NKG2A, an inhibitory C-type lectin-like receptor, forms heterodimers with CD94 and recognizes HLA-E molecules (62–64). The immunoglobulin-like transcript-2 (ILT-2) specific for HLA-G is also expressed by NK cells. It has been observed an inverse correlation between ILT-2 expression on T cells and clinical response in melanoma patients treated with oncolytic virus immunotherapy (65).

The discovery of inhibitory receptor-recognizing ligands other than MHC class I molecules such as TIGIT or the programed cell death-1 (PD-1) molecules constitute novel checkpoints in NK cell activation that requires further consideration (22, 31, 66–68). The PD-1/PD-L1 axis has been described as a checkpoint that regulates NK cell functions in tumor-bearing mice. Thus, blockade of PD-1/PD-L1 in nude mice resulted in anti-metastatic effect supporting the role of PD-1 on NK cell function (69).

Together with the expression level of MHC class I molecules on melanoma cells and the expression of MHC class I-specific inhibitory receptors on autologous NK cells, the expression of activating receptors on NK cells, and their ligands on melanoma are key actors in the final balance leading to an effective NK cell activation (9, 25).

## Melanoma Escape Mechanisms to Avoid NK Cell Cytotoxicity

Immune evasion by tumor cells through the down-regulation of MHC class I molecules to avoid CD8 T cell recognition constitutes a well-known mechanism used by melanoma (33). Melanoma loss of MHC class I expression increases its susceptibility to NK cells. As indicated above, the altered expression of HLA class I antigens is frequently found in melanoma (33), and several studies have shown that melanoma cells evolve down-regulating class I antigens to avoid being recognized by CD8^+^ T cells (34, 36). However, the analysis of the HLA class I antigen alterations in melanoma cell lines from ESTDAB showed that the most frequently observed phenotype is the down-regulation of HLA-B locus that is reversible after treatment with IFN-γ whereas the total lack of expression as a consequence of gene mutations or deletions leading to HLA heavy chain or β2m deficiency is only found in a minor group of samples (33). The bidirectional interaction between NK cells and melanoma cells induces changes in both effector and target cells (Figure 1D). It has been shown that melanoma immunoediting by NK cells make melanoma cells resistant to NK cell-mediated killing by increasing the expression of HLA class I molecules (70) and that blockade of HLA antigens with mAbs results in increased NK cell-mediated killing, indicating that HLA antigens expressed on melanoma cells interact with NK-inhibitory receptors avoiding NK cytotoxicity (71).

It has been also proposed that NK cell-mediated immunosurveillance against melanoma can generate immunoselection of melanoma cell variants with low expression of ligands for activating receptors that are resistant to NK cells (72). Thus, MICA and NCR ligand expression is lower in metastatic melanoma compared to primary melanoma lesions (6, 43). Shedding of soluble ligands for activating receptors constitutes another mechanism used frequently by melanoma cells to escape to the action of effector cells (25). Soluble NKG2D ligands MICA and ULBP2 are released by melanoma cells and can down-regulate the expression of NKG2D on effector cells. Thus, soluble ULBP2 was associated with lower survival in melanoma patients (38). NKG2D ligands can be released by ADAM protease-mediated shedding or secreted in exosomes with different functional outcomes (73). Shedding of B7-H6, a ligand for NKp30, by tumor cells has been recently described (74) also contributing to tumor escape from NK cells.

The down-regulation of NK cell-activating receptors has been described as an additional mechanism that contributes to tumor escape in cancer patients (25, 75–77). Thus, the decreased expression of NKp30 on NK cells from metastatic melanoma patients was associated with a reduced ability to kill melanoma cells (44). NK cells in stage IV melanoma patients displayed low levels of activating receptors that correlated with lower survival (20). IFN-γ released by NK cells induces indoleamine 2,3-dioxygenase (IDO) expression and prostaglandin E2 (PGE2) production by melanoma cells that inhibit NK cell function by down-regulating the expression of NKp30- and NKG2D-activating receptors further contributing to melanoma escape (78, 79).

T cell immunoreceptor with immunoglobulin and ITIM domains signaling after interaction with its ligands suppresses NK cell production of IFN-γ (67). In advanced melanoma patients, CD112 and CD155 were found upregulated in melanoma cells. In these patients, the expression of TIGIT either on CD8^+^ T cells or NK cells did not show significant differences compared with healthy donors whereas the expression of DNAM-1 on CD8^+^ T cells was down-regulated (66). These results suggest that inhibitory signaling through TIGIT can contribute to immune escape in melanoma.

Finally, suppression of NK cells by factors or cytokines secreted either by tumor cells or other cells in the tumor microenvironment such as myeloid derived suppressor cells (MDSCs) or macrophages can also contribute to immunoescape of cytotoxic cells (22).

All these mechanism together may contribute to the alterations of NK cell phenotype and function described in cancer patients.

## NK Cell-Based Immunotherapy in Melanoma

Different strategies of melanoma immunotherapy developed during the last decade focused on the use of checkpoints inhibitors or immune modulators, oncolytic virus therapy, cancer vaccines, adoptive T cell, and NK cell therapies and the use of cytokines (80). Many of those clinical trials are currently underway and include combined therapies. Here, we described those strategies focused on NK cell-mediated activation against melanoma or those immunotherapies that, although are not specifically directed to enhance NK cell function, may favor NK cell activation (Table 1).

**Table 1:** **NK cell-based immunotherapeutic strategies for melanoma**.

Category	Strategy	Start date–completion date	Melanoma patients	Phase/status	Identifier/reference
Autologous NK cells	LAK cells in combination with.IL-2 (i.v.)	1985	Seven metastatic melanoma	Phase I completed	Rosenberg et al. (92)
	Autologous NK cells combined with IL-2 (i.v.) and chemotherapy	2006–2009	Seven metastatic melanoma	Phase II completed	NCT00328861 Parkhurst et al. (93)
	Autologous NK cells and bortezomib (proteasome inhibitor)	2015 recruiting participants	Hematological and solid tumors including metastatic melanoma	Phase I	NCT00720785
Allogeneic NK cells	Allogeneic haploidentical NK cells	2004	10 metastatic melanoma	Phase I completed	Miller et al. (95)
	Allogeneic haploidentical NK cells (from PBMC) combined with chemotherapy	2009–2012	Refractory or relapsed melanoma	Phase I/II completed	NCT00846833
	Mismatched LAK followed by IL-2 (i.v.)	2009–2014	Malignant melanoma	Phase II completed	NCT00855452
NK cell line	NK92 cells		One metastatic melanoma	Phase I completed	Arai et al. (100)
Checkpoints/immune modulators	anti-KIR and anti-CTLA-4 anti-KIR and anti-PD-1	2012–2015 2015 recruiting participants	Advanced solid tumors Advanced solid tumors	Safety study Phase I	NCT01750580 NCT01714739

### Modulation of NK Cell Responses

There are different strategies to exploit the possibility to modulate NK cells in melanoma immunotherapy. The use of new forms of cytokine therapies or mAbs against tumor antigens can directly contribute to enhance NK cytotoxicity whereas immune checkpoints regulators constitute a novel immunotherapy strategy to modulate immune responses through their interaction with inhibitory receptors on immune cells.

#### Cytokines

Different cytokines have demonstrated a role in tumor immunity. Two cytokines have been approved by the Food and Drug Administration (FDA) for melanoma treatment as single agent: high doses of IL-2 for metastatic melanoma and IFN-α for the adjuvant therapy of Stage III melanoma based on the results obtained in clinical trials using high doses of IL-2 in metastatic melanoma patients (81) and IFN-α that demonstrated a significant benefit in relapse-free and overall survival of high-risk melanoma patients (82). Novel strategies have been developed such as bifunctional molecules consisting in cytokines fused to antibodies that allow the targeted delivery of the cytokines or the expression of cytokines in viral vectors or irradiated tumor cells for their use as vaccines. In addition, cytokines such as IL-2 or IL-15 are also used for the *in vitro* expansion of NK cells and T cells for adoptive transfer (83).

#### Checkpoint Blockade

As indicated before, one of the major checkpoints in NK cell activation is mediated by MHC class I-specific inhibitory receptors interacting with their ligands on target cells. Thus, blockade of this checkpoint constitutes an emerging area of research. Two NK cell checkpoint inhibitors lirilumab (anti-KIR mAb) and IPH2201 (anti-NKG2A mAb) are currently under revision. A safety study to analyze anti-KIR mAb in combination with ipilimumab (anti-CTLA4) (NCT01750580) is completed and a Phase I clinical trial of anti-KIR mAb in combination with anti-PD-1 is still recruiting patients (NCT01714739). IL-18 secretion by tumor cells upregulates PD-1 on NK cells (84). It has been shown that IL-18 secreted by tumor cells could elicits an expansion of NK cells overexpressing PD-L1 with immunoablative functions by reducing the number of mature NK cells and dendritic cells (DC) in a PD-L1-mediated manner, at least in the B16F10 melanoma model in mice (85). It has been suggested that the use of anti-IL-18 neutralizing antibodies in combination with anti-PD-1 mAb (nivolumimab) may bypass NK cell inhibition by PD-1 (22). Blocking several immune checkpoints can achieve synergistic anti-tumor effect with therapeutic benefits.

The clinical efficacy and pharmacological activity of anti-NKG2A mAb IPH2201 are going to be analyzed in clinical trials currently recruiting patients with squamous cell carcinoma of the oral cavity for an efficacy study of pre-operative use of IPH2201 (NCT02331875) or for a dose-ranging study of patients with high grade serious carcinoma of ovarian, fallopian tubes, or peritoneal origin (NCT02459301). The results of these trials may open new perspectives for melanoma treatment.

Increased tumor sensitivity to NK cells has been observed after treatment with proteasome inhibitors, doxorubicin or histone deacetylase inhibitors that upregulates the expression of NKG2D ligands, the secretion of proinflammatory cytokines, or the expression of TNF receptors. However, when combining these therapies with NK cell adoptive transfer, a strict control of NK cell function should be taken into account (22). In addition to the checkpoint blockade exerted by mAbs directed to receptors on cytotoxic cells or their ligands on tumors, mAbs may also act through ADCC or by redirected lysis of target cells.

#### Bispecific Killer Engagers

Novel strategies are in progress aimed to redirect NK cell cytotoxicity by CD16-directed bispecific and trispecific killer engagers (BiKEs and TriKEs respectively) constructed using one (BiKEs) or two (TriKEs) variable single-chain fragments against tumor-associated antigens. BiKEs and TriKEs trigger NK cell activation through CD16 (86). When combined with an inhibitor of ADAM17 to prevent CD16 shedding after NK cell activation, an enhancement of tumor cell lysis was observed (37, 87). The use of CD16-directed BiKEs has been limited so far to malignant hematological diseases.

### Adoptive NK Cell Therapy in Melanoma Patients

Optimal adoptive cancer immunotherapy should link both innate and adaptive immune responses. NK cells may contribute to the adaptive immune responses by favoring DC maturation and priming of T cells. The bidirectional crosstalk between NK cells and DC was demonstrated for the first time by Gerosa et al. in 2002 (88). NK cells activated by IL-2 or by mature DC directly induced DC maturation and enhanced DC ability to stimulate naïve CD4^+^ T cells. These effects were cell contact dependent, and IFN-γ and TNF secreted by NK cells also contributed to DC maturation (88). The interaction of NK cells and DC in the tumor microenvironment has shown to play a pivotal role in the induction of tumor-specific immune responses. However, tumor-induced immunosuppressive environment can deregulate the interactions of NK cells with DC (89, 90). Co-culture of DCs and lymphokine-activated killer (LAK) cells resulted in NK cell activation associated with enhanced inflammatory cytokine production and lysis of melanoma cells. LAK cell-mediated induction of DCs maturation has a significant effect on priming of anti-tumor CTLs (91).

#### Autologous NK Cells

Lymphokine-activated killer cells were used for the first time in melanoma patients by Roserberg et al. (92) showing complete remission in one patient with metastatic melanoma that lasted at least 10 months after combined therapy (LAK and IL-2).

Clinical trials of adoptive NK cell-based immunotherapy against melanoma are very limited. A Phase II trial (NCT00328861) completed in 2009 combined autologous NK cells with intravenous (i.v.) IL-2 and chemotherapy. Although no clinical effect was observed, the transferred NK cells persisted in the peripheral blood from 14 weeks to several months suggesting that combined therapy with antibodies could be beneficial (93).

Another trial using autologous NK cells combined with the proteasome inhibitor bortezomib is ongoing (NCT00720785). The use of bortezomib has been related to the upregulation of NKG2D ligands on tumor cells that may promote NK cell recognition and lysis of tumor cells (22).

Because, the expression of activating receptors on NK cells from tumor-bearing patients is frequently found down-regulated, the efficacy of autologous NK cells expanded *in vitro* is limited by the activating receptor phenotype of expanded NK cells that should be taken into consideration.

#### Allogeneic NK Cells

Few clinical trials using allogeneic NK cells for melanoma treatment have been reported usually combined with chemotherapy. It has been shown that NK cell activation of activating receptors together with administration of anti-tumor antibodies have substantial anti-cancer effects supporting that the combination of allogeneic NK cells and antibody therapy can be an efficient strategy in clinical trials (94). A phase I trial using allogeneic NK cells in 10 metastatic melanoma patients showed successful engraftment of NK cells. Four melanoma patients demonstrated stable disease after the first cell infusion but the disease progressed few weeks after a second infusion of NK cells. In the same trial, 5 of 19 poor prognosis AML patients achieved complete remission after NK cell infusion showing best results when KIR ligand mismatched donors were used (95). The role of haploidentical NK cell transfer was analyzed in a clinical trial (NCT00846833) in patients with refractory or relapsed malignant melanoma. A recent study analyzed the adoptive transfer of mismatched lymphocytes activated *in vitro* with recombinant human IL-2 (NCT00855452) for the induction of graft-versus-tumor effect in metastatic solid tumors including melanoma. The results of these trials have not yet been published.

#### Adoptive Transfer of NK Cell Lines

The difficulties of expanding large numbers of clinical grade NK cells (96) together with the lower transduction efficacy of primary NK cells are major limiting factors for their clinical application compared to NK cell lines. Further developments of viral vectors such as the alpharetroviral platform are required to fully exploit NK cells in cancer immunotherapy (97). It has been postulated that the use of NK cell lines that can be easily expanded *in vitro* could facilitate the development and standardization of protocols for the use of NK cells in therapy. The human NK cell line NK-92 (98) represents an alternative to donor-derived peripheral NK cells since it can be maintained *in vitro* and expanded to large numbers under good manufacturing practice (GMP) conditions for immunotherapy (99). The NK-92 cell line was evaluated in a Phase I trial in one metastatic melanoma patient that showed a minor response (100). The toxicity was low and this cell line was approved by the FDA for the treatment of melanoma. The possibility of engineered NK cell lines to express chimeric receptors has been also considered (101).

#### Chimeric Antigen Receptor-Modified NK Cells

A strategy to redirect NK cell cytotoxicity against melanoma is the use of chimeric antigen receptor (CAR)-modified NK cells. CARs consist of an external domain that specifically recognizes a given tumor antigen, linked with one or more intracellular signaling domains that trigger cytotoxic cell activation. NK cell lines, peripheral blood NK cells, and NK cells derived from human pluripotent stem cells can be engineered to express CARs. These CAR-transduced NK cells can specifically recognize and kill a variety of tumor targets expressing the surface target antigen [for review in Ref. (101, 102)]. It has been shown that the CAR-transduced NK92 cell line, NK-92MI-GPA7-zeta can recognize the melanoma-associated gp100 peptide in the context of HLA-A2, showing redirected killing of melanoma cell lines and primary melanoma (103). These results support the use of CAR engineering to redirect the specificity of NK cells to augment their cytotoxicity against tumors including refractory melanoma cells.

## Conclusion

Stimulation of the immune system has been considered a possible therapy for melanoma for many years. Experimental and clinical efforts have focused in exploring possibilities to use different elements of the adaptive and innate immune responses to control and eliminate melanoma cells. However, the heterogeneity of these tumors makes necessary a detailed analysis of the possible interactions between the melanoma and the immune system cells. NK cells are undoubted components within the anti-melanoma immunotherapy arsenal. The potential efficacy of NK cell-based immunotherapy in melanoma patients will rely on melanoma phenotype (expression of ligands for activating receptors and low expression of MHC class I molecules for the use of autologous NK cells), NK cell status (no exhausted, no senescent), NK cell phenotype (high level of NKG2D, NCRs and DNAM-1; CD16 expression for ADCC), microenvironment (proinflammatory versus inhibitory), NK cell crosstalk with other cell types (e.g., DCs, macrophages, MDSCs). The better understanding of the interactions between NK cells and melanoma will open the possibility to use combined strategies of checkpoints blockade and cytokine or activating receptor stimulation to enhance autologous NK cell cytotoxic capacity. These strategies should also be considered to modulate NK cell functionality in protocols of adoptive therapy against melanoma using autologous, allogeneic, or engineered *ex vivo-*expanded NK cells.

## Author Contributions

RT and RS designed the manuscript. RT, ED, and RS contributed to the writting and revised the manuscript.

## Conflict of Interest Statement

The authors declare that the research was conducted in the absence of any commercial or financial relationships that could be construed as a potential conflict of interest.
